# Nutritional Analysis of Five Wild Edible Vegetables Traditionally Consumed by the Orang Asli in Perak

**DOI:** 10.1155/2021/8823565

**Published:** 2021-06-08

**Authors:** Rachel Thomas Tharmabalan

**Affiliations:** School of Hospitality and Service Management, Sunway University, 5, Jalan Universiti, Bandar Sunway, 47500 Petaling Jaya, Selangor, Malaysia

## Abstract

The significance of wild edible plants may be traced back to antiquity, and methodological studies are the focus of present food movements to restore culinary traditions. Ethnobotanical appraisal was first done to determine the names and the significance of the five wild edibles used by the Orang Asli, which were *Erechtites valerianiaeolia* (Link ex Spring) DC, *Dendrocalamus asper* (Schult.) Backer, *Solanum nigrum* L., *Gomphandra quadrifida* (Blume) Sleumer, and *Pleocnemia irregularis* (C. Presl) Holttum collected from Sungkai, Perak in Malaysia. These wild edibles were then assessed for their proximate and mineral compositions. The present study revealed that the fiber content present in these wild edibles ranged from 2.7 to 12.5 g/100 g, whereas the protein content ranged from 1.8 to 6.8 g/100 g with *Gomphandra quadrifida* containing the highest amount of fiber and *Solanum nigrum* recording the highest protein content among the 5 wild edibles. In regard to the micronutreint content, iron was the highest in *Gomphandra quadrifida*, followed by *Pleocnemia irregularis*. Calcium and magnesium contents were the highest in *Solanum nigrum*. The results obtained in this study echo the urgency to conserve these plants in order to promote consumption as well as to improve the health and nutritional status of the Orang Asli.

## 1. Introduction

Human kind is experiencing very quick transformations in socio-economic expansion along with a rapid loss of biodiversity and emerging global health challenges. The recent 17 Sustainable Development Goals (SDGs) have been adopted by countries worldwide to target development challenges in terms of social, economic, and environmental issues that are plaguing both developed and developing countries worldwide, particularly in the agricultural sector. Being one of the top 10 megadiverse countries in the world, Malaysia has been experiencing a steady decline in the loss of valuable flora and fauna diversity due to rapid modernization and urbanization. Out of the 15000 species of vegetable plants available, only 300 species indigenous to the country have been used as food [1]. A recent report conducted by The Economist Intelligence Unit [2] showed that Malaysia has the highest obesity rate in Southeast Asia, and presently, noncommunicable diseases (NCDs) cause approximately 73% of total deaths in Malaysia. According to the findings from the Malaysian Adult Nutrition Survey 2003, it was published that fruit and vegetable consumption is still minimal among Malaysians [3].

According to Burlingame [4], there are at least one billion people who rely on wild edible plants for their sustenance. Wild edible plants are frequently found growing wild and free in marginal areas; therefore, they are regarded as a poor man's staple. However, they are crucial in local food and eating systems as they are an inherent part of local culture and are still used in traditional food preparations, and they are the focus of present food movements to restore culinary traditions. Over the past 5 years, AVRDC has analyzed the nutritional constituents of vegetables from around the globe only to find that wild edible plants possess much higher nutritional content, such as vitamins A, C, E, folate, iron, calcium, and antioxidant [5]. Several Amaranthus leafy vegetable species that have ethnobotanical, medicinal, and culinary importance, and exist both in wild and cultivated forms in native gardens, were evaluated for proximate compositions, such as protein, carbohydrates, fat, dietary fibers [6], and mineral compositions, such as K, Ca, Mg, Fe, Mn, Zn, and Mo [7]. These wild edibles tend to possess a much lower caloric content and glycemic index compared to commercially cultivated vegetables, thereby offsetting the negative effects of both malnutrition and obesity [8]. This, therefore, further advances the case that micronutrient-rich traditional plants should be brought back to present day diets to enhance the vitamin and mineral status among the population [9].

Rural populations of the world are the primary consumers and agents of preservation for these wild edibles. The Orang Asli, who are considered to be the first people, account for 13.9% of the 31 million people found in Malaysia [10]. Their identity has been long contested, though they are regarded as the first people to occupy Peninsular Malaysia [11]. The Orang Asli that inhabit remote areas still rely heavily on forest resources, wild edibles such as ferns, herbs, vegetables, tubers, and taro for their income and nourishment. Hence, to advocate utilization of these wild edible plants and preservation of their gene pool in Malaysia, the ethnobotanical importance of the wild edibles to the Orang Asli was recorded together with their proximate and mineral composition.

## 2. Materials and Methods

### 2.1. Study Area

**Figure 1:**
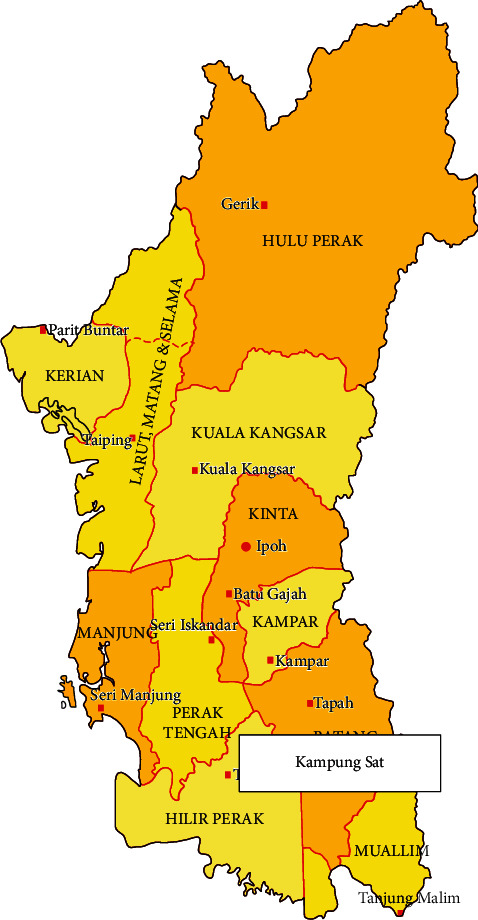
Map of Kampung Sat.

Kampung Sat is an Orang Asli settlement located at Sungkai, Perak (see Figure 1). The reason why this settlement was selected is due to the fact that it was located deeper in the jungle when compared to other Orang Asli settlements; hence, there were fewer detrimental environmental effects to be found. Kampung Sat's geographical coordinates are 4° 0′ 0^″^ N and 101° 18′ 36^″^ E and has an annual precipitation of 999.9 mm.

A survey of wild edible plants and their usage was conducted from 2017 to 2018 among the elders in the community utilizing ethnobotanical appraisal methods [12]. Relevant information such as their location, general facts about the habitat, and local plant names was also documented alongside pictures to show their morphological features.

The plant species selected for nutritional evaluation were then arranged in terms of their species name, local name, family name, parts used, consumption method, and alleged medicinal properties.

### 2.2. Proximate Analysis

Vegetables were washed to remove visible dirt under running potable water to ensure samples were safe and suitable for analysis. To obtain a constant value for moisture content, samples were then dried in an oven at 60°C for 24 h. Samples were then ground into powder and stored for later use.

The proximate analysis, which encompassed moisture, ash, crude fat, crude protein, fiber content, carbohydrate, and energy of the sample, was determined according to the standard method described by the Association of Official Analytical Chemists [13]. Samples were placed in a muffle furnace at 550 ± 15°C for 24 h to gravimetrically determine the ash content. The Soxhlet method was employed to determine the fat content, whereas protein was estimated using the Kjedahl method and then multiplied by 6.25. The amount of carbohydrates present was calculated based on difference by deducting the gross sum of percent of moisture, ash, crude protein, total fat, and dietary fiber from 100. The sample analysis was all carried out in triplicate.

Energy was calculated based on the formula reported by [14]:

(1) $$\begin{array}{c} \text{Energy }\left(\text{kcal per }100\text{ g}\right)=\left(\text{Protein }\left(\text{g}\right)\times 4\frac{\text{kcal}}{\text{g}}\right)+\left(\text{lipid }\left(\text{g}\right)\times 9\frac{\text{kcal}}{\text{g}}\right)+\left(\text{carbohydrates }\left(\text{g}\right)\times 4\frac{\text{kcal}}{\text{g}}\right). \end{array}$$

The data of wild edible plants used in this study were expressed as g/100 g, and energy was expressed as kJ/100 g.

### 2.3. Mineral Determination

Six widespread minerals, which include calcium (Ca), potassium (K), iron (Fe), magnesium (Mg), zinc (Zn), and copper (Cu), were determined using the atomic absorption spectrophotometric method [13]. Wet ashing method was employed, whereby 12 mL of the concentrated oxi-acidic mixture of nitric acid HNO_3_ and perchloric acid HClO_4_ (4 : 1) was added to 0.5 g of samples. A graph was plotted for absorption (nm) against concentration (*μ*g/mL) of standard solution with known concentrations to determine the mineral content under optimized conditions. The average values were calculated based on triplicate reading. The amount of element in each gram of the sample was calculated as follows:

Amount of element, ppm (*μ*g/g) = (*μ*g/mL) × *F*/g sample, where *F* = (mL original dilution × mL final dilution)/mL aliquots if original 100 mL is diluted. The data were expressed as mg/100 g

## 3. Results and Discussions

### 3.1. Ethnobotanical Observations

The Orang Asli at Kampung Sat possess a wealth of indigenous knowledge in regard to these wild edibles in terms of their common usage, dietary habits, and medicinal values. All these 5 wild edibles are easily accessible and readily available for consumption, as much of the jungle is still left intact. The ethnobotanical information is presented in Table 1.

**Figure 2:**
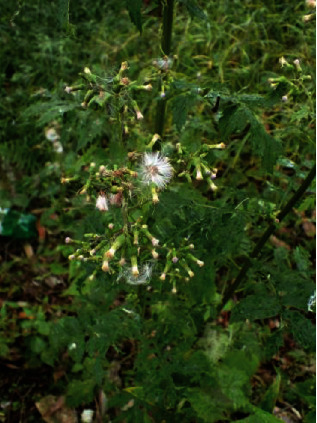
*Erechtites valerianiaeolia* (Link ex Spring) DC.

*Erechtites valerianiaeolia* (Link ex Spring) DC. (Figure 2) is part of the Asteraceae family. It is considered to be the most diverse family of flowering plants on Earth, with more than 23000 species [15]. It is found in both East and West Malaysia and can be found growing in many different habitats, in even unfavourable conditions. The Orang Asli use this wild edible to treat simple ailments such as fever, tonsils, and skin problems.

**Figure 3:**
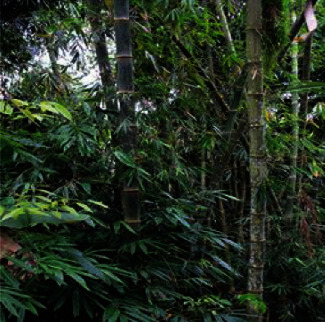
*Dendrocalamus asper* (Schult.) Backer.

Bamboo, which originates from the *Poaceae* family, is native to Africa, Asia, and Central and South America and is widely related to indigenous culture and knowledge [16]. It is regarded as the fastest growing and most economically important plant in the world, with more than 1500 uses, such as in housing, utensils, furniture, handicraft, paper, agricultural activities, boats, bicycles, clothes, and among many others [17]. There are reportedly 70 species of bamboo found in both East and West Malaysia [18]. *Dendrocalamus asper* (Schult.) Backer shoots (Figure 3) of young bamboo plants are traditionally used as food by many Orang Asli as well as raw material for building houses, furniture, and cooking materials.

**Figure 4:**
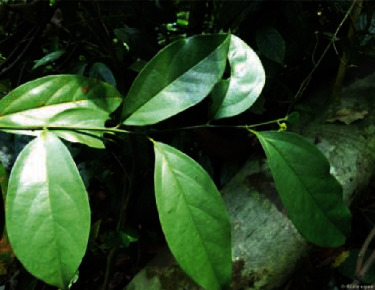
*Gomphandra quadrifida* (Blume) Sleumer.

*Gomphandra quadrifida* (Blume) Sleumer (Figure 4) is a wild and native shrub and tree (5 m in height) under the Stemonuraceae family, found in the Orang Asli villages. Known as “bern-go” by the Orang Asli, its waxy leaves consist of a midrib that is very obvious at the lower part of the tree and are the ultimate food of choice for the Malayan tapirs [19]. For the Orang Asli women, they are usually given a mixture of the leaves to be drunk to help them recover faster after delivery.

**Figure 5:**
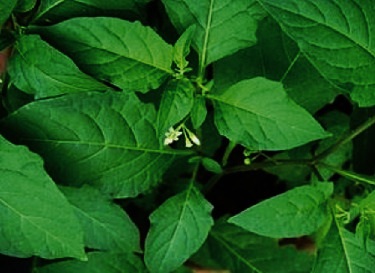
*Solanum nigrum* L.

The “kera,” also known as *Solanum nigrum* L. (Figure 5), is from the Solanaceae family and is considered a weed which grows in arable lands and nitrogen rich soil in tropical and subtropical climates. The parts of the plant that can be utilized include the leaves, shoots, and fruits which have been used in many marginally poor countries as medicinal herbs, ancient family plants, and vegetables [20]. Traditionally, the leaves and fruits have been used to treat diabetes and high blood pressure [21]. The unripe fruits, however, are toxic in nature, as they contain exceeding amounts of solanine. Among the Orang Asli folks, they have started planting these vegetables in their own backyard. The stem of a “kera” is semithick, and when cooking this vegetable, the shoots and leaves are boiled in water, with the water being replaced one or two times to prevent the consumption of toxins. *Solanum nigrum* is commonly used to treat high blood pressure, according to many Orang Asli informants, as they have been utilizing it to treat this ailment for the past three decades. The leaves are ground and made into a paste and then variously used.

**Figure 6:**
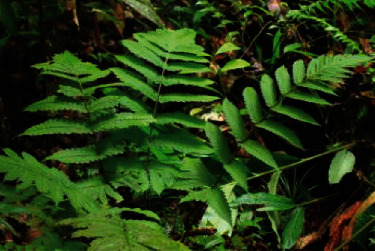
*Pleocnemia irregularis* (C. Presl) Holttum.

*Pleocnemia irregularis* (C. Presl) Holttum (Figure 6), also known as “ber-negy” among the Orang Asli, are considered native plants in Southeast Asia and are part of the Dryopteridaceae family. Younger leaves are preferred and can both be eaten raw or cooked. If eaten raw, they are usually mixed with other vegetables due to their astringent taste. There are no reports of domestication, as it grows wild and free in the villages of the Orang Asli and propagates through spores. Ong et al. [22] reported that *P. irregularis* has been used among the Temuans to treat diarrhoea, skin disease, and weak muscles. The Orang Asli are not worried about the loss of this species as it easily grows near the settlements.

### 3.2. Proximate Composition

Based on the analysis done (Table 2), wild edible vegetables had conspicuously higher fiber, mineral, and protein content as compared to commercial vegetables, such as “Kang Kung” (*Ipomoea aquatica*), “Bok Choy” (*Brassica campestris L*.), and “Gai Lan” (*Brassica oleracea*). These traditional plants also have very high moisture content (72.3-89.7 g/100 g) thus giving them a much higher energy density. This is ideal, because it reduces the amount of calories, yet contributes to satiety. Vegetables like *Solanum nigrum* and *Gomphandra quadrifida* have a significantly higher fiber content compared to *Dendrocalamus asper* and *Pleocnemia irregularis*. The fat and carbohydrate contents of *Erechtites valerianiaeolia* were 0.2 g/100 g and 2.5 g/100 g, respectively. The proximate compositions obtained in this study were corroborative to the findings of leafy vegetable amaranths [23]. Barreira et al. [24] also noted that the *E. valerianifolia* obtained in Brazil would make a good food source based on its availability as well as its ability to meet the daily recommended nutrient intake, hence potentially reducing nutritional deficiencies among the population.

The average protein content among wild edible plants is 1.8-6.8 g. This is considerably low, as most of the plants have incomplete amino acids. However, when comparing with commonly consumed commercial vegetables in Malaysia such as “Kang Kung” (*Ipomoea aquatica*), “Bok Choy” (*Brassica campestris L*.), and “Gai Lan” (*Brassica oleracea*) (3 g/100 g, 1.5 g/100 g, and 1.2 g/100 g) [25], these wild edibles have a higher protein content. Protein is essential for tissue growth and repair as well as regulating various body functions. The wild edibles used in the study were relatively low in fat (below 0.7 g), therefore contributing 12.4-61.9 kcal per 100 g for every of wild vegetable consumed. The fiber content is the highest in *Gomphandra quadrifida*, contributing 44.64% to the RDA needs as compared to 0.9% found in *Ipomoea Aquatica*, 2.5% *Brassica Campestris L*., and 8% in *Brassica Oleracea* [25]. The carbohydrate content varied from less than 0.1 g in *Pleocnemia irregularis* to 7.7 g in *Gomphandra quadrifida*.

### 3.3. Mineral Composition

Minerals are inorganic compounds that are required to perform body functions that are vital for life and are essential for physiological well-being. The calcium content found in these wild edibles ranged from 85.5 to 354 mg/100 g, whereas the highest iron content was seen in *Gomphandra quadrifida* 5.9 mg/100 g. The potassium content ranged from 265 to 655 mg/100 g, with the highest in *Solanum nigrum*. In regard to the magnesium content, *Solanum nigrum* showed 79 mg/100 g followed by *Pleocnemia irregularis* with 63 mg/100 g.

The Orang Asli have been using *Solanum nigrum* to treat hypertension, and this has been validated by Schilling et al. [26] who reported on *Solanum*'s effectiveness. Recent medical studies have also proven its effectiveness in reducing blood glucose and lipid levels in the body and that it can potentially be used to reduce cardiovascular problems in diabetic patients [27].

Minerals like potassium, magnesium, calcium, and iron work together to help reduce blood pressure. Based on the quantitative results, *Solanum nigrum* is high in all of these four nutrients. Potassium helps to maintain salt equilibrium and muscle function in the body, whereas magnesium acts as a transporter for both calcium and potassium and helps blood vessels relax. A recent study by the National Institutes of Health reported that older men who consumed magnesium on a regular basis have much lower blood pressure. Also, higher potassium consumption has been linked to a reduction in the risk of death from ischemic heart attack when compared to individuals with potassium deficiency [28]. Calcium helps to control the contraction and relaxation of blood vessels, which is vital in maintaining healthy blood pressure. Apart from being scientifically validated to be a super nutrient, calcium decreases many diet-related diseases like cardiovascular disease and osteoporosis and also reduces the risk of colorectal cancer among males and ovarian and breast cancer among females and premenopausal woman. The high phytochemical content found in traditional plants acts as antioxidants and protects against free radical damage which causes cancer [29]. Dairy products, which are higher in saturated fat, tend to promote bone fracture and cardiovascular disease. The exact opposite can be said about plant-based sources of calcium like “bok choy” and soybeans, as they also supplement diets with vitamins C and K and the minerals potassium and magnesium. It has been reported by Darwina and Wan Puteh [30] that the prevalence of hypertension among the Orang Asli community stood at 30.8%, which is 1.4% lower than the national average reported in the 3rd National Health Morbidity Survey. Also, the rate of overweight and obese individuals stood at 30.0% and 17.7%, respectively, which is as high as the general Malaysian population [31]. Hence, consuming *Solanum nigrum* could potentially reduce the prevalence of hypertension among them. The nutritional studies of these plants have proven and exceeded expectations as these vegetables have all the necessary micronutrient content to ensure nutritional security.

## 4. Conclusion

From this study, wild edibles have a rich nutrient profile and provide a good source of nutrients for the diets of the Orang Asli. Therefore, the wild edibles analyzed in this study can be considered potential vegetables as they provide the essential nutrients needed in sufficient amounts to promote health and well-being among the Orang Asli. The leaves of *Solanum nigrum L.* have greater potentiality compared to other wild edibles due to their medicinal value and nutritional content. As these wild edibles are readily available in Kampung Sat, it would be of immediate value to the Orang Asli and local residents to generate better evidence of the health and nutrition attributes, especially in terms of toxic substances, found in these plants, and to quantify the benefits of the diversity of the indigenous and underutilized foods to their livelihoods and to ensure that such information gathered is put to use widely to increase their well-being and to aid in the conservation of biodiversity for future generation.

## Figures and Tables

**Table 1:** Ethnobotany of wild edible plants selected for nutritional analysis.

Species name	Local name	Family name	Parts used	Cooked/raw	Alleged medicinal properties
*Erechtites valerianiaeolia* (Link ex Spring) DC.	Ber-pa	Asteraceae	Leaves	Cooked	Skin problems, fever, and tonsillitis
*Dendrocalamus asper* (Schult.) Backer	Rabo	*Poaceae*	Shoots	Raw and cooked	Increase appetite
*Gomphandra quadrifida* (Blume) Sleumer	Bern-go	Stemonuraceae	Leaves	Cooked	Recovery after child birth
*Solanum nigrum* L.	Kera	Solanaceae	Leaves, fruits	Cooked	High blood pressure
*Pleocnemia irregularis* (C. Presl) Holttum	Ber-negy	Dryopteridaceae	Fronds	Raw and cooked	Diarrhoea, skin infection

**Table 2:** Proximate composition (g/100 g), energy (kcal/100 g), and minerals and trace minerals (mg/100 g) of four different species of edible wild vegetables and its Recommended Dietary Allowance (source: [25]).

Traditional wild plants	*Erechtites valerianiaeolia* (Link ex Spring) DC.	*Dendrocalamus asper* (Schult.) Backer	*Gomphandra quadrifida* (Blume) Sleumer	*Solanum nigrum* L.	*Pleocnemia irregularis* (C. Presl) Holttum
Moisture (g/100 g)	83.5 ± 0.51	89.7 ± 1.43	72.3 ± 0.99	85.2 ± 0.76	87.7 ± 1.31
Ash (g/100 g)	2.8 ± 0.76	9.65 ± 0.54	10.9 ± 0.12	8.9 ± 0.43	14.2 ± 0.13
Fiber (g/100 g)	3.8 ± 0.24	2.7 ± 0.44	12.5 ± 0.35	7.3 ± 0.64	2.8 ± 0.82
Fat (g/100 g)	0.2 ± 0.11	0.3 ± 0.24	0.7 ± 0.14	<0.1 g	<0.1 g
Protein (g/100 g)	1.8 ± 0.71	3.8 ± 0.38	6.2 ± 0.24	6.8 ± 0.45	3.1 ± 0.86
CHO (g/100 g)	2.5 ± 0.11	5.1 ± 0.14	7.7 ± 0.52	0.4 g ± 0.12	<0.1 g
Energy (kJ/100 g)	19 ± 0.12	38.3 ± 0.98	61.9 ± 0.45	28.8 ± 0.12	12.4 ± 0.13
Calcium (mg/100 g)	85.5 ± 1.42	135 ± 1.31	325 ± 0.67	354 ± 0.14	107 ± 0.29
Iron (mg/100 g)	2.2 ± 0.11	0.42 ± 0.03	5.9 ± 0.14	3.6 ± 0.23	4.9 ± 0.15
Potassium (mg/100 g)	525 ± 1.62	265 ± 0.51	564 ± 1.42	655 ± 0.76	576 ± 0.41
Magnesium (mg/100 g)	15.6 ± 0.23	13.6 ± 0.46	31.8 ± 0.11	79 ± 1.2	63 ± 0.43

## Data Availability

The author confirms that the data supporting the findings of this study are available within the article.
